# Stability in metabolic phenotypes and inferred metagenome profiles before the onset of colitis-induced inflammation

**DOI:** 10.1038/s41598-017-08732-1

**Published:** 2017-08-18

**Authors:** M. Glymenaki, A. Barnes, S. O’ Hagan, G. Warhurst, A. J. McBain, I. D. Wilson, D. B. Kell, K. J. Else, S. M. Cruickshank

**Affiliations:** 1 Faculty of Biology, Medicine and Health, University of Manchester, Manchester, UK; 2 Shimadzu Corporation, Manchester, UK; 3 School of Chemistry, University of Manchester, Manchester, UK; Manchester Institute of Biotechnology, University of Manchester, Manchester, UK; 4 Infection, Injury and Inflammation Research Group, Division of Medicine and Neurosciences, University of Manchester and Salford Royal Hospitals NHS Trust, Salford, UK; 5 Section of Biomolecular Medicine, Division of Computational and Systems Medicine, Department of Surgery and Cancer, Imperial College, London, UK

## Abstract

Inflammatory bowel disease (IBD) is associated with altered microbiota composition and metabolism, but it is unclear whether these changes precede inflammation or are the result of it since current studies have mainly focused on changes after the onset of disease. We previously showed differences in mucus gut microbiota composition preceded colitis-induced inflammation and stool microbial differences only became apparent at colitis onset. In the present study, we aimed to investigate whether microbial dysbiosis was associated with differences in both predicted microbial gene content and endogenous metabolite profiles. We examined the functional potential of mucus and stool microbial communities in the *mdr1a* ^−/−^ mouse model of colitis and littermate controls using PICRUSt on 16S rRNA sequencing data. Our findings indicate that despite changes in microbial composition, microbial functional pathways were stable before and during the development of mucosal inflammation. LC-MS-based metabolic phenotyping (metabotyping) in urine samples confirmed that metabolite profiles in *mdr1a* ^−/−^ mice were remarkably unaffected by development of intestinal inflammation and there were no differences in previously published metabolic markers of IBD. Metabolic profiles did, however, discriminate the colitis-prone *mdr1a* ^−/−^ genotype from controls. Our results indicate resilience of the metabolic network irrespective of inflammation. Importantly as metabolites differentiated genotype, genotype-differentiating metabolites could potentially predict IBD risk.

## Introduction

Inflammatory bowel disease (IBD), which includes Crohn’s disease (CD) and ulcerative colitis (UC), is associated with an overreacting immune response and alterations in gut microbial communities referred to as dysbiosis^1, 2^. Dysbiosis in IBD is characterized by decreased bacterial diversity and an imbalanced microbial composition^3–5^. Enterobacteriaceae are enriched, whereas clades IV and XIVa Clostridia and members of Bacteroidetes are reduced during IBD development^4–6^.

Perturbations in microbial gene content abundance and expression occur as a consequence of the underlying IBD-associated dysbiosis and have been reported in IBD and experimental models of colitis^7–11^. Microbial gene functions related to oxidative stress resistance and nutrient transport are reportedly increased in colitis at the expense of basic biosynthetic processes such as amino acid biosynthesis, thus indicating alterations in energy metabolism within the intestinal microbiota during IBD^7–9^. To date, studies on the gene functional profile of gut microbial communities have focused on changes during active inflammation or remission and thus they may be a secondary effect of inflammation, while there is little information on potential changes preceding inflammation.

Changes in the taxonomic composition of microbial species or their activities may impact on the metabolic processes in the colon, leading to an altered metabolite profile^12–15^. Metabolite profiling studies using a range of biofluids such as faecal water, urine or serum have been used to differentiate IBD patients from healthy individuals^12, 15–18^. In particular, urinary metabolites reflect endogenous metabolites produced by host metabolism as well as metabolic products of bacterial metabolism and host-bacterial co-metabolism such as hippurate^14, 19^. Hence, the analysis of metabolites in urine offers a relatively non-invasive means by which systemic changes seen before and during IBD can be investigated.

We have previously shown that changes in gut microbiota profiles in the mucus but not in faeces precede onset of inflammation in colitis-prone mice^20^. We utilized the *mdr1a* ^−/−^ spontaneous model of colitis, because it has an intact immune system, requires no physical intervention for colitis to develop^21, 22^ and polymorphisms of this gene are linked with increased susceptibility to UC in humans^23, 24^. Based on our previous findings, we hypothesized that shifts in mucus microbial communities may correlate with changed function and altered metabolite profiles. To assess the functional profile of microbial communities in both mucus and faeces, we performed an *in silico* analysis of 16S rRNA gene sequencing data coupled with reference genomes. As a subset of the encoded functions of microbial communities is expressed at any given time, we further employed a metabonomic approach using urine samples with the aim of detecting potential metabolite changes that might strongly influence the host-microbiota crosstalk prior to inflammation. Our findings indicate the stability of microbial gene coding potential and endogenous metabolites prior to the development of mucosal inflammation and suggest resilience of metabolism before and during disease outbreak.

## Materials and Methods

### Maintenance of animals

*Mdr1a* ^−/−^ mice (FVB.129P2-*Abcb1a* ^*tm1Bor*^ N7)^25^ and control FVB mice (Taconic Farms, NY, USA) were crossbred to generate F1 heterozygotes, which in turn were crossbred to generate F2 littermate controls. Mice were given autoclaved standard chow and sterile acidified water (pH = 3.2) *ad libitum*. *Mdr1a* ^−/−^ and wild-type (WT) control males were maintained under co-housing conditions to ensure shared microbiota. All animals were kept under specific, pathogen-free (SPF) conditions at the University of Manchester and experiments were performed according to the regulations issued by the Home Office under amended ASPA, 2012.

### Histology and colitis scoring

Distal colon tissue was fixed, paraffin-embedded and stained with haematoxylin and eosin, and with alcian blue dye for colitis scoring as previously described^20^. In brief, the sum of scores for crypt length elongation (score 0–4), goblet cell depletion (score 0–4), muscle wall thickness (score 0–4), inflammatory cell infiltration (score 0–4) and destruction of architecture (score 0 or 3–4) was calculated as detailed in an earlier study from our group on *mdr1a* ^−/−^ mice^20^.

### Isolation of bacterial genomic DNA

Bacterial genomic DNA was isolated from faecal and mucus samples as previously reported^20^ and extracted using the QIAamp^®^ DNA Stool Mini Kit (Qiagen, Manchester, UK) with an additional bead beating step.

### 16S rRNA gene sequencing analysis

The V3 and V4 variable regions of the 16S rRNA gene were PCR amplified for sequencing on the Illumina MiSeq platform according to manufacturer’s guidelines as previously described^20^. Sequences were submitted to European Bioinformatics Institute (EBI) for quality filtering^26^ and were further processed using the Quantitative Insights Into Microbial Ecology (QIIME) pipeline v.1.9.0^27^. They were assigned to operational taxonomic units (OTUs) using a closed-reference OTU picking strategy^28^ and taxonomically classified using the Greengenes database filtered at 97% identity^29, 30^.

PICRUSt (phylogenetic investigation of communities by reconstruction of unobserved states) was then applied on the Greengenes picked OTU table to generate metagenomic data and derive KEGG (Kyoto Encyclopaedia of Genes and Genomes) Orthology gene abundance data^31^. KEGG Orthology gene family abundances were summarized at a higher hierarchical level at pathway-level categories for easier biological interpretation. Non-microbial categories such as ‘Organismal Systems’ and ‘Human Diseases’ were excluded from further analysis. Beta diversity of rarefied KEGG pathway data was calculated using the Bray-Curtis distance metric and visualized using Principal Coordinate Analysis (PCoA) in Matlab (MathWorks, MA, USA). KEGG pathway abundance data between groups were compared using group_significance.py in QIIME^27^. Metagenomic data were also analysed using STatistical Analysis of Metagenomic Profiles (STAMP) software^32^. To examine PICRUSt’s predictive accuracy, the weighted nearest sequenced taxon index (NSTI) values were calculated (Supplementary Table S1).

### Urine sample collection and preparation

Urine samples were collected from mice at designated time points in clean autoclaved cages or by injection in the bladder during culling and stored at −80 °C (n = 18 WT 6 weeks, n = 18 *mdr1a* ^−/−^ 6 weeks, n = 17 WT 18 weeks, n = 12 *mdr1a* ^−/−^ 18 weeks). Urine preparation was performed according to a previously published method^33^. In brief, samples were thawed at room temperature and centrifuged at 10,000 g for 5 min. Then, 10 μL urine was diluted with 40 μL water (or by 1:4 v:v in samples of less than 10 μL). 5 μL of each diluted sample was injected onto to the analytical column. Quality control (QC) samples were prepared by pooling aliquots of 10 μL of each sample^34^.

### Ultra high performance liquid chromatography- mass spectrometry (UHPLC-MS) metabolite analysis

For analysis 5 μL aliquots of each sample (maintained at 4 °C in the autosampler) were injected for separation by reversed-phase UHPLC, using gradient elution, with a Nexera LC system (Shimadzu Corporation, Kyoto, Japan) on to the analytical column, an Acquity HSS T3 1.8μm C18 (100 × 2.1 mm) (Waters Corporation, Milford, USA) at a flow rate of 0.4 mL/min, with the column maintained at 40 °C. Chromatography was performed via gradient elution using a binary solvent system according to a previously published method^35^. Solvent A comprised water containing 0.1% formic acid and solvent B was acetonitrile containing 0.1% formic acid. The gradient conditions were: 2% B (2 min), to 35% B (12 min), to 100% (18 min) held to 23.5 min, re-equilibration time was 5 min. Samples were analysed by LC-MS using a quadrupole ion-trap time-of-flight mass spectrometer (LCMS-IT-TOF, Shimadzu Corporation, Kyoto, Japan) equipped with an electrospray source in both positive and negative ionisation mode (polarity switching time of 100 msecs). The mass range measured was m/z 60–1250 in MS mode. Mass calibration was carried out using a trifluoroacetic acid sodium solution (2.5 mmol/L) from 50 to 1000 Da. Other instrument parameters included: ion source temperature of 250 °C, heated capillary temperature of 230 °C, electrospray voltage of 4.5 kV, electrospray nebulization gas flow was 1.5 L/minute, detector voltage 1.7 kV. Data acquisition and processing used software LCMS Solution (version 3.8, Shimadzu Corporation, Kyoto, Japan).

### LC-MS data analysis and processing

Profiling Solution software (version 1.1, Shimadzu Corporation, Kyoto, Japan) was used to create an aligned data array of retention time, m/z and intensity data for both positive and negative ion data as previously described^36^. Sample data acquisition was performed in four acquisition events to provide optimum sensitivity in the lower mass range whilst not saturating in the higher mass range: positive mode m/z 60–200, positive mode m/z 140–1250, negative mode m/z 60–200, negative mode m/z 140–1250. Data were combined into a single data array containing 3129 ions in which no data point was excluded. Profiling Solution software generated an aligned data array of both positive and negative ion MS data, which was subsequently exported to SIMCA-P (version 14, Umetrics, MKS Instruments Inc., Sweden) for principal components analysis (PCA). Following noise reduction thresholding, a data array was processed using SIMCA-P and scaled to unit variance (the base weight is computed as 1/sdj, with sdj is the standard deviation of variable j computed around the mean). No variable was excluded in this analysis. Metabolite features were statistically tested for their quantitative significance by considering the reproducibility of the ion signal in the pooled QC sample. Profiling Solution processing parameters included: 15mDa ion bin m/z tolerance, 0.2 min ion bin RT tolerance, noise threshold 100000. Pooled QA/QC parameters: 80% ions required from all QC samples, better than 30% relative standard deviation (RSD) peak area precision, better than 5% RSD retention time precision.

### Multivariate statistics on LC-MS data

LC-MS data of urine samples were subjected to multivariate statistical analysis using KNIME^37–39^ and R (http://cran.r-project.org). Pre-processing involved removing QC and “singleton” data, followed by application of a correlation filter for removal of correlated features (threshold = 0.98) and Z -scores normalization (Z = (x−µ)/σ). In addition to PCA, multivariate regression was applied, as it correlates independent variables in matrix X (i.e. metabolite data) to corresponding dependent variables in matrix Y (i.e. groups, classes)^40^. Partial least squares (PLS) regression and random forests (RF) regression was used to construct predictive regression models for better discrimination of sample groups^41, 42^. PLS- linear discriminant analysis (PLS-LDA), a specific form of PLS regression, was used in the case of dependent categorical variables. The performance of the PLS-regression model was tested using cross-validation with the ‘leave one out’ method. All data were used for training in the model, which does not rule out the potential for over-fitting of the data.

### Feature permutation method

To identify mass ions, also referred to as features, that contributed to differential classification of sample groups, mass ions were permutated and classification cross entropy was calculated. An RF classifier was used in a simplified method as follows. First, the mean cross entropy and sigma for 100 repeats of the unpermuted RF data were calculated and then each feature column was permuted and the cross entropy was recalculated. Repeats on each permutation were performed in a loop to derive the mean cross entropy. The difference between the mean permutated result and mean unpermuted result was measured and if the difference was found to be greater than 1.96 * sigma, it was considered significant with a P value equal to 0.95. The Storey multiple comparison correction method was further applied^43^. When a feature is permuted, the cross entropy will increase if it is significant meaning that classification will get worse.

When there are relatively few samples and noisy data, machine-learning methods can often pick out noise as features. To deal with this issue, re-pre-processing the data offers a way of systematic error removal. Thus, data were re-processed with a 0.3 min tolerance in retention time alignment and a noise thresholding set to 1,000,000 was used. Nevertheless, this approach still generated a small number of noise ions, so a second stage analysis was applied that generated the so-called Chromatogram Matrix in which generic peak integration parameters were applied to all chromatographic peaks from ions identified in the spectral matrix. PCA and regression analysis of these data also led to similar conclusion as the initial analysis before re-processing.

### Metabolite identification

To identify biologically significant components, high mass accuracy MS and MSn fragment ion information was used to determine the most likely candidate formula (mass accuracy of LCMS-IT-TOF typically better than 5ppm). Authentic chemical standards were also purchased for confirmation of metabolite identity. Endogenous creatine and histidine were used as internal standard compounds for data normalisation. Data acquisition and processing used software LCMS Solution (version 3.8). Analysis of pooled QC data of known endogenous metabolites showed acceptable precision with better than 30% RSD (peak area ratio) for most of the known candidate marker metabolites specifically targeted for examination, with the exception of leucine and isoleucine that are challenging to separate by reversed-phase chromatography (Supplementary Table S2).

### Statistical analysis

Statistical analysis was performed using R and GraphPad Prism 6 (GraphPad software, CA, USA). The vegan package in R was also used for carrying adonis test. Normally distributed data were analysed by unpaired t test. Data that did not exhibit a normal distribution were analysed using Mann-Whitney test or the nonparametric Kruskal-Wallis test with Dunn’s posttest as appropriate to the number of comparisons being made. P < 0.05 was considered as statistically significant (*P < 0.05, **P < 0.01). All P-values were corrected for multiple hypothesis testing using the Benjamini- Hochberg false discovery rate (FDR) method unless otherwise stated^44^.

### Ethical approval

Procedures were performed in accordance with the United Kingdom Animals (Scientific Procedures) Act of 1986, and conformed to the Directive 2010/63/EY of the European Parliament. Ethical permission was obtained from the University of Manchester Animal Welfare and Ethical Review Board and performed under a Home Office approved grant.

## Results

### Gut microbial predicted functional profiles remain stable prior to and during inflammation

**Figure 1:**
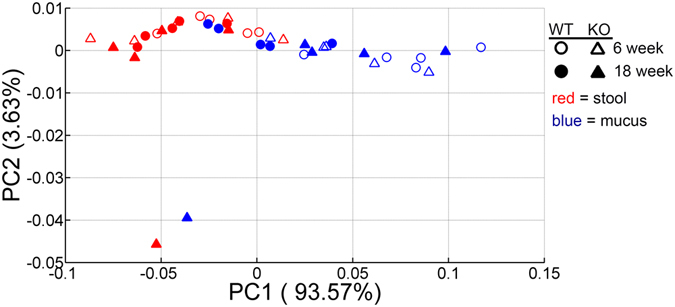
Stability of the microbial functional potential prior to the development of colitis. Microbial genes were inferred by PICRUSt from 16S rRNA gene sequences and assigned to functional pathways as organized in KEGG database. Principal Coordinate Analysis (PCoA) plot of Bray-Curtis distance comparing microbial functional profiles between *mdr1a* ^−/−^ and WT littermates at 6 and 18 weeks showed clustering of samples according to sampling location (i.e. mucus and stools). Bray-Curtis distances were calculated based on KEGG pathway abundance values. WT mice are shown in circles and knockout (KO) mice in triangles; open symbols correspond to 6 weeks whereas filled ones to 18 weeks. Mucus is depicted in blue and stools in red.

All mice were assessed for indications of colon inflammation by histology and qPCR for inflammatory genes as published previously^20^. At 6 weeks of age, although all mice were histologically normal with no expression of Interferon γ, microbial composition was altered in the mucus but not in the stools of *mdr1a* ^−/−^ mice in contrast to WT controls at 6 weeks of age, approximately 12 weeks before signs of intestinal inflammation are detected^20^. However, by the later time point of 18 weeks of age, signs of inflammation were apparent and changes in microbial composition were detected in both the mucus and stools of *mdr1a* ^−/−^ mice^20^. To investigate the microbial functional potential in mucus and stools prior to, and during, the development of colitis, we applied PICRUSt on 16S rRNA gene amplicon sequencing data^31^. PICRUSt infers the approximate gene content of detected phylotypes. PICRUSt-predicted gene families represented by KEGG Orthology groups were binned into KEGG metabolic pathways to assess the similarity of the functional state of the microbiome. PCoA analysis using Bray-Curtis distance demonstrated that samples were separated mainly according to their location (i.e. mucus or stool), which accounted for 93.57% of the overall variation between samples (Adonis test; R2 = 0.52, P = 0.001) (Fig. 1). Samples were further stratified according to their sampling location (i.e. mucus or stool) to examine possible differentiation across age without the confounding effect of location (Supplementary Fig. S1a,b). We observed no clear clustering of samples depending on age especially in stools (Adonis test; R2 = 0.102, P = 0.145). Age contributed to the separation of mucus samples (Adonis test; R2 = 0.24 P = 0.021). In a similar mode, genotype was not able to separate samples obtained at 6 and 18 weeks from mucus and stools (Supplementary Fig. S2a,d).

The relative abundance of KEGG metabolic pathways was similar between WT and *mdr1a* ^−/−^ mice before the onset of intestinal inflammation, irrespective of microbial habitat, mucus or stools (Supplementary Fig. S3a,b). Functional pathways’ abundance remained unmodified even when signs of inflammation started (Supplementary Fig. S4a,b). Thus, functional categories implicated in metabolism, genetic information processing, environmental information processing and cellular processes presented a steady pattern across time independent of genotype. Notably, a great proportion of the microbial functions were poorly characterized or unclassified. Differences in KEGG metabolic pathways were only observed between mucus and stool microbial communities (Supplementary Fig. S5). Collectively, these data suggest that microbial functional potential is remarkably stable prior to and during inflammation in the gut.

### Metabolic phenotyping revealed no differences in identified IBD marker metabolites

Dysbiosis in IBD is reportedly associated with disruption of host-microbiota dialogue with an impact on host immune system and metabolism resulting in loss of homeostasis^45^. To examine putative effects of previously reported altered microbial composition on the metabolite profiles of *mdr1a* ^−/−^ mice versus WT controls, we performed untargeted LC-MS analysis of urinary samples collected at 6 and 18 weeks so as to determine metabolic phenotypes. We selected urine, as it provides a pool of endogenous host metabolites that also reflect bacterial metabolism^14, 19^. Within this untargeted approach, we took advantage of the method to also look for changes in endogenous metabolites that had been highlighted in previous publications to be significantly changed in human and murine IBD studies in order to determine if there were similar differences in *mdr1a* ^−/−^ mice before and during the onset of inflammation (Supplementary Table S2). Hippurate has long been considered a significant marker metabolite, as it is affected by microbiota changes^46^ and is also known to be decreased in UC and CD patients^12, 17^.

**Figure 2:**
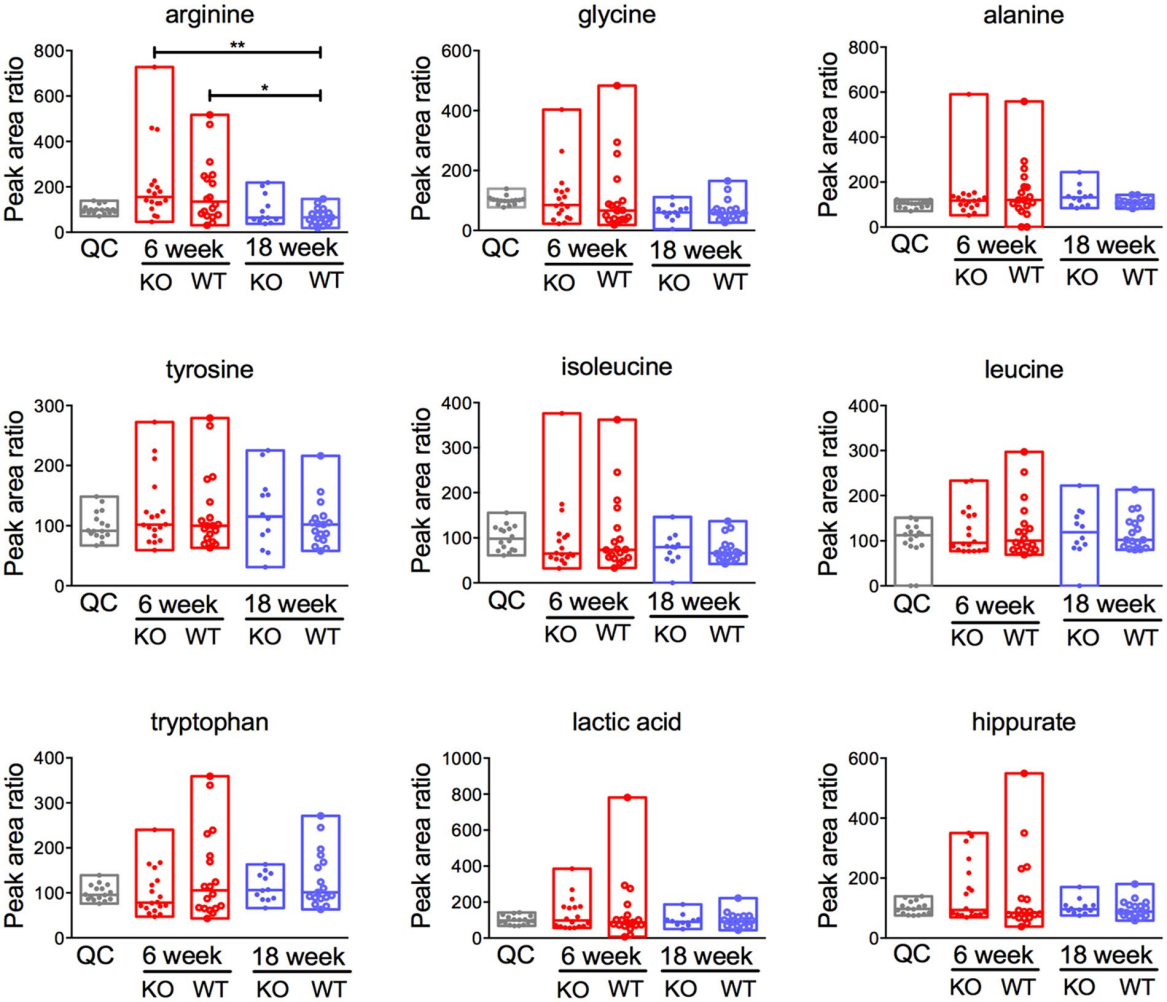
IBD marker metabolites in urine samples from WT and *mdr1a* ^−/−^ mice at 6 and 18 weeks. No differences were identified in the quantities of metabolites when comparing samples of the same genotype across time or between WT and KO samples at 6 weeks or 18 weeks. Creatine was used as an internal control. N = 18 WT 6 weeks, N = 18 *mdr1a* ^−/−^ 6 weeks, N = 17 WT 18 weeks and N = 12 *mdr1a* ^−/−^ 18 weeks. The median is shown as a line and bars capture the minimum and maximum. *P < 0.05; **P < 0.01 as determined by Kruskal-Wallis test with Dunn’s multiple comparisons test.

To account for sample-to-sample differences in dilution, endogenous creatine or histidine were used as internal standards compound for all metabolites. No significant differences in urinary metabolites including hippurate could be identified between 6 and 18-week WT and *mdr1a* ^−/−^ mice except for arginine, which was reduced over time (Fig. 2). Further to this, relative concentrations of previously reported influential metabolites such as glutamine, phenylalanine and succinate^17, 47–50^ were comparable between WT and *mdr1a* ^−/−^ mice at 18 weeks, despite the appearance of signs of inflammation in *mdr1a* ^−/−^ mice (Supplementary Fig. S6).

### Metabolite profiling discriminates colitis-prone genotype prior to the onset of inflammation

**Figure 3:**
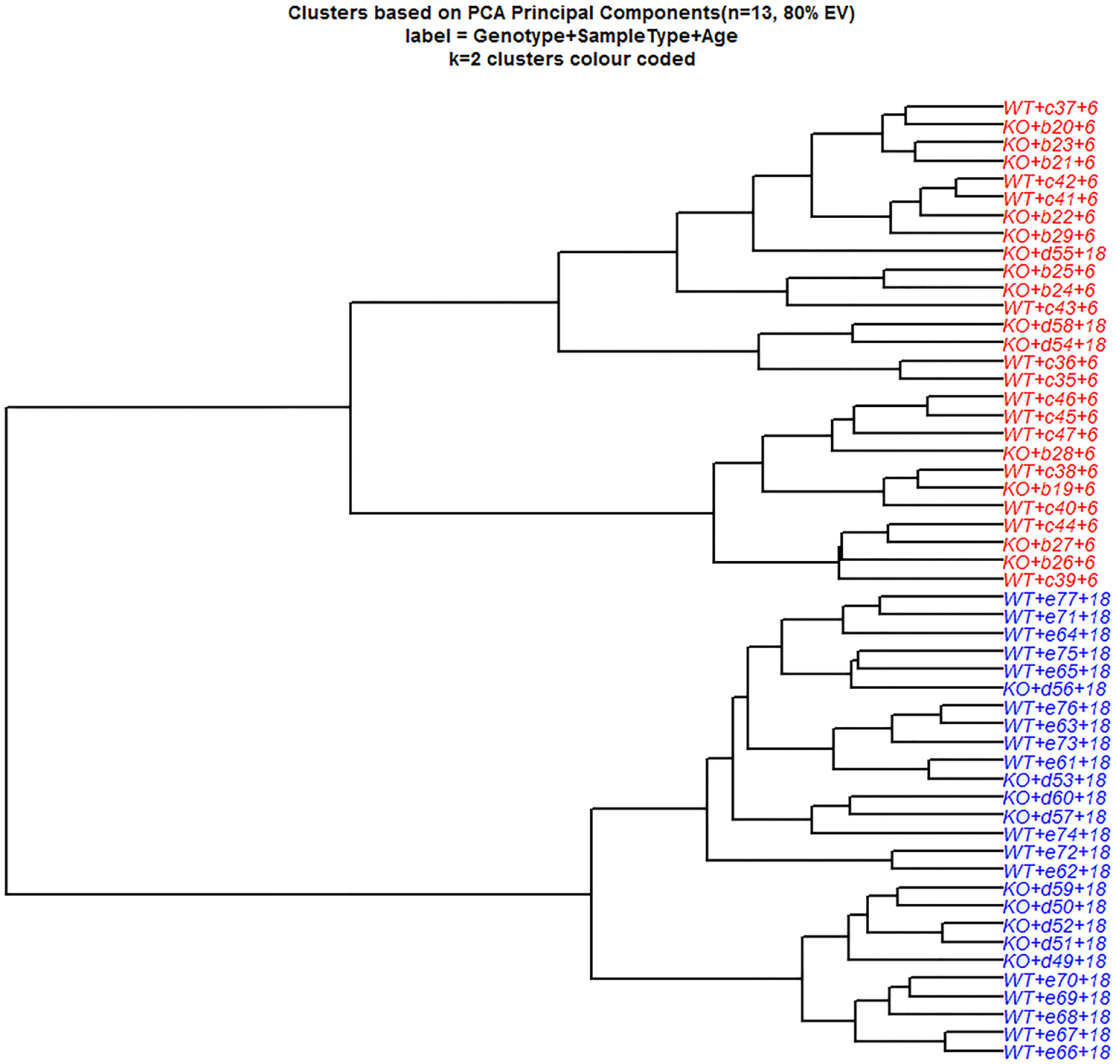
Stability of urinary metabolite profiles in colitis-prone *mdr1a* ^−/−^ animals during onset of inflammation. Principal Components Analysis (PCA) of mass ions measured by LC-MS for urine samples from *mdr1a* ^−/−^ mice and WT littermate controls at 6 and 18 weeks of age. Clustering of PCA data separated samples according to age, which accounted for 41.7% of the total variance. Data are shown in a dendrogram.

Since analysis centred on previously reported metabolites takes into account only a tiny fraction of the urinary metabolite profile, we also performed an untargeted analysis on all of the data acquired to examine a wider pool of metabolites. PCA analysis was first carried out on LC-MS-derived data to provide an overview of the variations between WT and *mdr1a* ^−/−^ mice at 6 and 18 weeks (Fig. 3). PCA revealed a pattern of variation associated with age reflected by differential clustering of 6-week and 18-week samples, which explained 41.7% of the variation.

**Figure 4:**
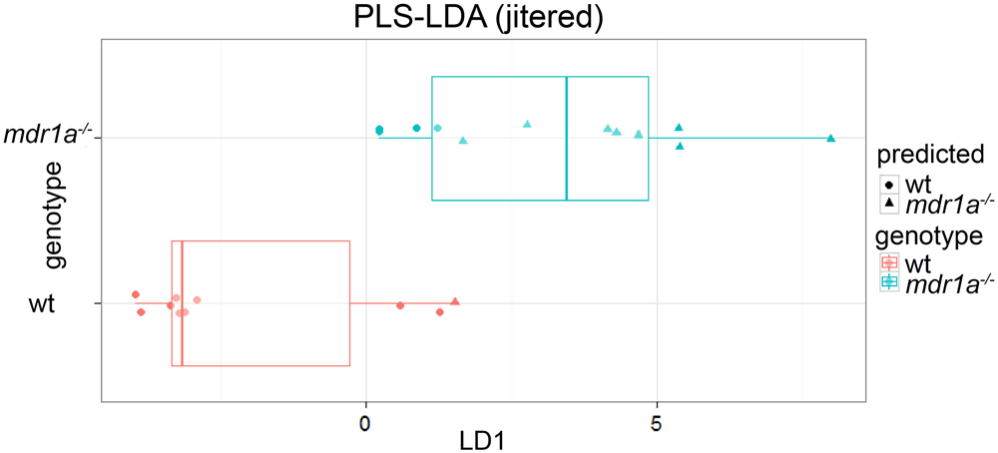
Partial least squares-linear discriminant analysis (PLS-LDA) discriminates urinary metabolite profiles from *mdr1a* ^−/−^ and WT mice based on genotype. Boxplot of genotype predictions for WT and *mdr1a* ^−/−^ mice, where the central rectangle spans the first quartile to the third quartile with median shown as a line; whiskers above and below the box represent the maximum and minimum respectively. True *mdr1a* ^−/−^ are in blue, predicted *mdr1a* ^−/−^ are triangles; true WT are in red, predicted WT are in circles. Therefore, blue circles are false negatives and red triangles are false positives.

PCA is an unsupervised method for assessing variation among samples that ignores the correlation between LC-MS mass ions and sample characteristics such as age, genotype or colitis scoring. For this reason, we applied PLS-LDA (a supervised classification method) that considers the correlation of LC-MS variables to the class membership of samples (for example WT or *mdr1a* ^−/−^ in the case of genotype) to maximize their separation. When PLS-LDA was undertaken, the LC-MS profiles from WT and *mdr1a* ^−/−^ mice were differentially clustered (Fig. 4) with an accuracy of 80% in predictive regression models (Table 1). To discover the metabolites which contributed to the discrimination of urinary metabolic profiles between WT and knockout (KO) animals permutational analysis was performed. The mean cross entropy changes on feature permutation found differences based on genotype; however, study limitations prevented assigning metabolite biomarkers from these data. Specifically, LC-MS peaks of permuted features using the whole dataset as input showed that that the three ion signals coming as significant (F2_186: m/z = 415.2563, RT = 10.182; F2_91: m/z = 302.2206, RT = 7.599; and F2_111: m/z = 319.1925, RT = 7.036) were of low spectral intensity (Supplementary Fig. S7a,b). Therefore, confidence in mass accuracy was not sufficient to assign these mass ions to known metabolites. As 6-week animals had higher variation in the targeted metabolites than 18-week animals, which were more closely clustered, and since genotype appeared to be the main discriminating factor, subsequent permutation analysis was performed including only 18-week animals. Permutation analysis based on 18-week LC-MS derived data identified four ions (F2_128: m/z = 355.0955, RT = 4.164; F2_182: m/z = 413.2144, RT = 9.195; F2_90: m/z = 299.1478, RT = 4.798; and F2_91: m/z = 302.2206, RT = 7.599) as discriminatory (Supplementary Fig. S8a). However, subsequent analysis revealed that the first ion detected was absent from the profiling data array due to data misalignment and thus data were re-pre-processed (see also Supplementary materials and methods). Feature importance was not flagged as significant when looking at q-values using the re-pre-processed data (Supplementary Fig. S8b).

**Table 1:** Prediction statistics for the PLS-LDA^a^ obtained from LC-MS data.

row ID	TP^b^	FP^c^	TN^d^	FN^e^	Recall	Precision	Sensitivity	Specificity
*Mdr1a* ^−/−^	8	1	9	4	0.67	0.89	0.67	0.90
WT	9	4	8	1	0.90	0.69	0.90	0.67

^a^5 latent variables were used for the PLS-LDA model, ^b^True Positives, ^c^False Positives, ^d^True Negatives, ^e^False Negatives. F-measure for *mdr1a* ^−/−^ is 0.76 and for WT is 0.78.Overall accuracy is 0.77 and Cohen’s kappa 0.55. All LC-MS mass data were used for the PLS-LDA model. No sample was excluded from the analysis.

PLS regression and RF regression were used to build models in order to identify changes in metabolite profiles that could predict varying scores of colitis (Supplementary Fig. S9). This showed that metabolic variations were not correlated with different colitis scores, as they deviated from the identity line. These data suggest that the aforementioned metabolic changes are independent of ongoing inflammation, but rather they are related to genotype. As *mdr1a* ^−/−^ mice are prone to colitis development as they age^21, 22^, differences observed in metabolites not affiliated with inflammation could indicate a predictive risk of IBD.

## Discussion

IBD is associated with an altered gut microbiota composition, which may also be translated to an altered metabolic activity in the gut^8, 51^. Here we show that microbial functional pathways remain stable before the onset of intestinal inflammation in colitis-prone mice. In our previous findings we observed changes in the overall taxonomic composition of mucus in *mdr1a* ^−/−^ mice 12 weeks prior to inflammation, and in both mucus and faeces during colitis development at 18 weeks^20^. Therefore, our results indicate that changes in predicted microbial functional gene potential do not accompany altered bacterial composition. Interestingly, previous studies have demonstrated that different assemblages of microbial communities may converge to similar metabolic functions in the gut^52, 53^. Differences in the clustering of microbial gene pathways were only observed between mucus and stool microbial communities, which are known to differ in microbial composition^8, 54^.

Our data indicate a stable pattern in the inferred functional profiling of microbial communities in *mdr1a* ^−/−^ mice relative to their WT counterparts at time points before and during the start of inflammation. Previous functional profiling studies of the gut microbiome have mainly focused on established disease^7–10^. Microbial gene families involved in nutrient transport and oxidative stress resistance were increased during active colitis^7–9^, whereas energy metabolism and amino acid biosynthesis pathways were reduced^8, 9^. Additionally, microbial functions contributing to bacterial pathogenesis were enriched^8, 9^. These functional changes suggest an adaptation of multiple microbes to accommodate the environmental stressors present in the inflamed gut. A recent study of the colonic microbiota from healthy individuals carrying *FUT2* gene polymorphism, a CD risk allele, revealed alterations at both the compositional and functional level of the gut microbiome, which were accompanied by sub-clinical inflammation in the intestinal mucosa^55^.

We inferred gene content of the gut microbiome using PICRUSt, which gives an accurate but approximate prediction based on reference microbial genomes^31^. However, potential bias may be introduced from 16S rRNA reads that failed to map to reference OTUs and the lack of availability of a sufficient number of reference genomes with will then impact on PICRUSt’s predicted metagenome accuracy. In our study, 93.56% of 16S rRNA sequences mapped to reference OTUs, but NSTI scores were high (>0.15) indicating low availability of closely related reference genomes, which is also illustrated in the proportions of poorly characterized and unclassified gene functions. The relatively high NSTI values suggest there will be a lower prediction accuracy of the gene content of the 16S rRNA samples derived from mucus and faeces based solely on microbial composition. Furthermore, this type of analysis does not provide information about the expression of encoded microbial gene functions, thus we further analyzed metabolites to complement the inferred microbial functional profiling observations.

To shed light on putative metabolic changes, we performed a metabolite analysis of urine samples. The untargeted metabolic phenotyping approach showed that the relative concentrations of metabolites previously reported to change in human and murine IBD studies were similar between WT and *mdr1a* ^−/−^ mice at both 6 and 18 weeks with the exception of arginine, which was significantly decreased with age. PCA analysis of untargeted metabolites also showed an age-dependent variation. These findings are consistent with previous studies on IBD murine models and specifically on *IL-10* ^−/−^ mice that have reported age-related effects on the metabolome^49, 56^. Hippurate, which is consistently reduced in UC and CD^12, 17^, displayed comparable concentrations among groups. Predictive models were also not able to correlate metabolite profiles to colitis scoring. Taken together, our results suggest that the overall metabolic state of the animal was not affected even at the onset of inflammation; however we cannot exclude the possibility that changes will occur once severe inflammation develops. Indeed, clinical studies of IBD in humans that already have established disease and exhibit disease symptoms have shown changes in metabolite profiles^12, 15–18^. Further to this, studies in colitic IL-10^−/−^ mice as well as dextran sulphate sodium (DSS)-colitis induced mice have shown that differences in metabolite profiles were more profound once inflammation had progressed and colitis had developed^49, 50, 56–58^. Therefore, metabolic changes observed in these studies follow the development of intestinal inflammation and as a consequence the disruption of host-microbiota homeostasis^14, 59^. Here we looked at stages before the development of overt mucosal inflammation and found no metabolic changes between WT and *mdr1a* ^−/−^ mice.

Our results show that metabolite profiles classified differentially according to genotype. Metabolic composition differed between WT and m*dr1a* ^−/−^ mice, which progress to colitis with aging^21, 22^. However, low spectral intensity in conjunction with study limitations prevented the accurate assigning of these differentiating features to known metabolites. In support of genotype-related metabolic changes, a study on dietary supplementation has shown that *mdr1a* ^−/−^ mice fed a control diet had lower amounts of short chain fatty acids (SCFAs) and higher quantities of lactic and succinic acid in their faeces^60^. Of note, butyrate is a SCFA that contributes to the maintenance of immune tolerance^61, 62^. Further work to ensure that sufficient material is obtained to enable full structural characterisation and identification of the potential metabolic biomarkers detected in the urinary profiles of these mice will be required as part of any effort to translate this work to humans.

Collectively, the findings of the present study demonstrate that the activity of microbial communities and urinary metabolites are remarkably stable in *mdr1a* ^−/−^ mice in the face of ensuing gut inflammation despite changes in mucus microbial community composition. The biochemistry of the host and its associated gut microbiota seems to remain unaffected at stages preceding full disease manifestation possibly due to function redundancy in microbial and host metabolic pathways or underlying subtle changes with a minimal effect. However, metabolite profiles differed depending on genotype, indicating alterations in the metabolic network unrelated to inflammation. The fact that changes in metabolites previously associated with gut inflammation were not observed suggests that these genotype-affiliated metabolites could constitute a predictive risk of IBD in at least a subset of patients carrying MDR1A polymorphisms and warrants further investigation to identify the metabolites and pathways involved as part of further studies on this mouse model of colitis. Additional experiments evaluating reportedly altered microbial functions such as nutrient transport and oxidative stress resistance in fecal and mucus microbial communities before and during inflammation should be undertaken to support the assertion of stability in functional profiles. Longitudinal studies on other murine models of IBD and patients would be necessary before translating such research finding to humans with IBD.

## Electronic supplementary material

Supplemental data and methods
