# Supplementary material for: Stability in metabolic phenotypes and inferred metagenome profiles before the onset of colitis-induced inflammation
Source: Sci Rep. 2017 Aug 18;7:8836. doi: 10.1038/s41598-017-08732-1 (PMC5562868; doi:10.1038/s41598-017-08732-1)
Supplement: Supplementary file 1 — Supplemental data and methods [file 41598_2017_8732_MOESM1_ESM.pdf]

# Supplementary materials and methods

## Stability in metabolic phenotypes and inferred metagenome profiles before the onset of colitis-induced inflammation

M. Glymenaki, A. Barnes, S. O' Hagan, G. Warhurst, A.J. McBain, I.D. Wilson, D.B. Kell, K.J. Else, S.M. Cruickshank*

*To whom correspondence should be addressed.

Email: sheena.cruickshank@manchester.ac.uk

## Supplementary materials and methods

### Isolation of bacterial genomic DNA

Bacterial genomic DNA was isolated from faecal and mucus samples as previously shown [1]. Distal colon tissue was excised, opened up and washed in sterile PBS for the removal of luminal contents. Mucus was scraped for the collection of bacteria that inhabited the outer mucus and the inner adherent layer. DNA extraction was performed using the QIAamp® DNA Stool Mini Kit (Qiagen, Manchester, UK) with an additional bead beating step [2].

### Real-time reverse transcriptase (RT)-PCR

RNA extraction from proximal colon tissue samples was performed using TRIsure (Bioline, London, UK) in accordance with the manufacturer's instructions. RNA was reverse- transcribed using the Bioscript reverse trancriptase (Bioline). The cDNA was subjected to Real-time RT-PCR using the Power SYBR Green Master Mix (Applied Biosystems, supplied by Thermo Fisher Scientific, Paisley, UK) as previously described [1] in order to examine the expression of Interferon γ (primer F: 5'-GCGTCATTGAATCACACCTG-3', R: 5'-ACCTGTGGGTTGTTGACCTC-3'). Gene expression values were normalized based on GAPDH expression (primer F: 5'-CCCACTAACATCAAATGGGG -3', R: 5'-TCTCCATGGTGGTGAAGACA -3') for each sample.

### 16S rRNA gene sequencing analysis

The V3 and V4 variable regions of the 16S rRNA gene were PCR amplified for sequencing on the Illumina MiSeq platform according to manufacturer's guidelines as previously reported [1]. Illumina sequencing generated paired-end reads of 300bp in each direction. After demultiplexing, overlapping paired-end reads were joined using SeqPrep (http://github.com/jstjohn/SeqPrep) and submitted to European Bioinformatics Institute (EBI) for quality filtering [3]. The quality-filtering process included removal of reads with low quality ends (i.e. ambiguous leading/trailing bases), removal of reads where the proportion of ambiguous bases is higher than 10% and removal of reads with length less than 300bp [3]. Thus sequencing errors (i.e. singletons/doubletons) shall be removed from downstream processing. After passing a

filter for prokaryotic rRNA reads, sequences were further processed using the Quantitative Insights Into Microbial Ecology (QIIME) pipeline v.1.9.0 [4]. They were assigned to operational taxonomic units (OTUs) using a closed-reference OTU picking strategy [5] and taxonomically classified using the Greengenes database filtered at 97% identity [6,7]. A resulting OTU table was generated giving the OTU abundances in each sample with taxonomic identification for each OTU.

PICRUSt (phylogenetic investigation of communities by reconstruction of unobserved states) was then applied on the Greengenes picked OTU table to generate metagenomic data and derive KEGG (Kyoto Encyclopaedia of Genes and Genomes) Orthology gene abundance data [8]. The PICRUSt algorithm infers the approximate gene content of detected phylotypes (OTUs) based on a database of reference genomes. It basically transforms OTU counts generated by closed reference picking OTU strategy in QIIME into predicted gene family counts. The OTU table was initially corrected by normalizing by predicted 16S rRNA copy number for each OTU. Inferred KEGG gene abundances were summarized at a higher hierarchical level at pathway-level categories for easier biological interpretation. Non-microbial categories such as ‘Organismal Systems’ and ‘Human Diseases’ were excluded from further analysis. Beta diversity of rarefied KEGG pathway data was calculated using the Bray-Curtis distance metric and visualized using Principal Coordinate Analysis (PCoA) in Matlab (MathWorks, MA, USA). KEGG pathway abundance data between groups were compared using group_significance.py in QIIME [4]. Metagenomic data were also analysed using STatistical Analysis of Metagenomic Profiles (STAMP) software [9].

To examine PICRUSt’s predictive accuracy, the weighted nearest sequenced taxon index (NSTI) values were calculated. NSTI values represent the average branch length that separates each OTU in a sample from a sequenced reference genome, weighted by the abundance of that OTU in the sample [8]. Therefore, NSTI values summarize the extent to which OTUs in a sample are related to sequenced genomes. Low NSTI values indicate higher prediction accuracy.

**Multivariate statistics on LC-MS data**

LC-MS data of urine samples were subjected to multivariate statistical analysis using KNIME [10-12] and R (http://cran.r-project.org). Principal components analysis (PCA) was performed to provide an overview of the samples' distribution and identify potential patterns of variation. Data pre-processing involved removing QC and "singletons", followed by application of a correlation filter for removal of correlated features (threshold = 0.98) and Z -scores normalization ($Z = (x - \mu)/\sigma$). PCA calculates principal components, which are linear combinations of the initial variables (i.e. metabolites), explaining most of the variation within the dataset [13]. Score plots of PCA analysis were generated and each sample was represented in the new coordinate space. The corresponding loading plot for each principal component was also produced to indentify which mass ions contribute to patterns of variation as observed in the scores plot.

Multivariate regression was applied for data analysis, as it correlates independent variables in matrix X (i.e. metabolite data) to corresponding dependent variables in matrix Y (i.e. groups, classes) [14]. This approach aims to maximize the covariance between X and Y matrices by finding a linear relation. Thus, partial least squares (PLS) regression was used to construct predictive regression models for better discrimination of sample groups [14,15]. Y variables (i.e. sample groups) were predicted from the model based on a reduced number of factors (PLS components) [15]. The performance of each model was tested using cross-validation with the 'leave one out' method. All data were used for training in the model, which potentially does not rule out potential over-fitting of the data.

Random forests (RF) regression was further applied to build prediction models [11]. RF is a classification method, in which many decision trees are constructed using different sets of random variables and samples [16,17]. An advantage of this method is that it is robust to over-fitting and no data transformation (such as standardization) is required prior to the analysis [15]. The original data are split in training and test sets using bootstrapping (with replacement), whereby training sets are useful for tree construction and test sets for calculation of prediction accuracy.

A specific form of PLS regression is PLS- linear discriminant analysis (PLS-LDA). PLS-LDA, a supervised classification method, relates LC-MS variables to the class

membership of samples to maximize the separation of samples according to their classification. Therefore, PLS-DA handles dependent categorical variables compared with PLS regression that uses dependent continuous variables [15]. The PLS-LDA model chosen was the one that gave the lowest mean classification error rate for 20 "bootstrap samples". The misclassification matrix describes the number of correctly predicted samples, the specificity, sensitivity and the positive and negative predictive values of the model. Score plots were generated and mass ions responsible for differences between classes were searched for by inspection of regression vectors and variable importance in projection (VIP) scores. However, as the VIP threshold is hard to define, a way of settling this is by looking at separate validation data versus threshold. As the data were insufficient for this, a features' permutation approach was followed.

**Feature permutation**

LC-MS peaks of permuted features using the whole dataset as input showed that that the three ion signals coming as significant (F2_186: m/z = 415.2563, RT = 10.182; F2_91: m/z = 302.2206, RT = 7.599; and F2_111: m/z = 319.1925, RT = 7.036) were of low spectral intensity (Fig. S7A-B). Therefore, confidence in mass accuracy was not sufficient to assign these mass ions to known metabolites. As 6-week animals had higher variation in the targeted metabolites than 18-week animals, which were more closely clustered, and since genotype appeared to be the main discriminating factor, subsequent permutation analysis was performed including only 18-week animals.

Permutation analysis based on RF classification of 18-week LC-MS samples identified four ions as discriminatory (F2_128: m/z = 355.0955, RT = 4.164; F2_182: m/z = 413.2144, RT = 9.195; F2_90: m/z = 299.1478, RT = 4.798; and F2_91: m/z = 302.2206, RT = 7.599) (Fig. S8A). The first ion detected (i.e. F2_128: m/z = 355.0955, RT = 4.164) was however absent from the profiling data array due to data misalignment (the spectral ion matrix was binned with a 15mDa tolerance and 0.2min). As a consequence of data misalignment, data were re-processed with a 0.3min tolerance in retention time. Setting data alignment tolerances aims to enable alignment of chromatographically resolved ions from different data files; setting a tolerance too narrow can cause misalignment through systematic changes during batch acquisition, conversely setting tolerances too wide can cause incorrect ion

binning due to isomers (particularly lipid species), which can be binned incorrectly with too high a tolerance. The same can also happen with mass tolerance misalignment however with good LC separation applied it is rare for retention time and mass tolerance issues to occur at the same time. In the case of this data, analysis of peak area data after reprocessing revealed that retention time misalignment had occurred and the ion found as significant was in fact the same in all sample groups and was not significant. Following re-processing the data with wider retention time tolerance may have slightly changed the PCA plots, regression and classification results, so they were reprocessed and recalculated but no change was found.

The spectral matrix data processing parameters were set to "de-isotope" the data array to avoid duplication of ions, however some isotopes can still remain in the matrix if isotope intensities are not sufficiently aligned. The statistical analysis, although very powerful, may have been finding features within LC-MS noise; thereby data were re-processed again applying a noise thresholding set to 1,000,000 (previously set to 100,000). Nevertheless, this approach still generated a small number of noise ions, so a second stage analysis was applied that generated a Chromatogram Matrix in which generic peak integration parameters were applied to all peaks identified in the spectral matrix.

When we have relatively few samples and noisy data, machine learning methods can often pick out noise as features and as a result this warrants cautiousness about claims made for the contribution of certain ions. To deal with this issue, re-pre-processing data offers a way of systematic error removal. Another permutation technique including permutation of the target class a few hundred times was also used, as in that way the link between features and target class would break, allowing us to determine the likelihood of getting a good classification accuracy "by accident"; additionally it may give a similar insight into accidental feature ranking. A caveat in this method is that issues such as de-isotoping errors and mass binning errors would probably manifest as systematic errors and typical statistical methods such as permutation may not be of help.

PCA and regression analysis of the newly pre-processed data also led to similar conclusion as the initial analysis before re-processing. Classification accuracy of this

data was similar to that found for the full data set, however feature importance was not flagged as significant when looking at q-values; the best q-value was very poor at 0.48 (Fig. S8B). Since using the 18-week data only results in the number of cases being halved, it is possible that the power of the analysis was compromised.

**Supplementary Table S1. NSTI values to evaluate PICRUSt accuracy.**

| Group | Mean | Standard deviation (SD) | Age (weeks) | Genotype | Location |
|---|---|---|---|---|---|
| Group A | 0.290 | 0.023 | 6 | *mdr1a*$^{-/-}$ | Stool |
| Group B | 0.267 | 0.021 | 6 | wt | Stool |
| Group C | 0.229 | 0.028 | 6 | *mdr1a*$^{-/-}$ | Mucus |
| Group D | 0.203 | 0.024 | 6 | wt | Mucus |
| Group E | 0.262 | 0.045 | 18 | *mdr1a*$^{-/-}$ | Stool |
| Group F | 0.285 | 0.019 | 18 | wt | Stool |
| Group G | 0.222 | 0.026 | 18 | *mdr1a*$^{-/-}$ | Mucus |
| Group H | 0.260 | 0.027 | 18 | wt | Mucus |

**Supplementary Table S2. Previously published significant endogenous metabolites in human UC / CD studies and in murine IBD models. These metabolites were detected in our analysis and confirmed by authentic standard analysis.**

| Metabolite | [M+H]+ | RT[a] (min) | QC[b] % RSD[c] | Formula | Sample type | Reference[d] |
|---|---|---|---|---|---|---|
| Arginine | 175.1190 | 0.55 | 19% | C6H14N4O2 | Urine | [18] |
| Glycine | 76.0393 | 0.65 | 14% | C2H5NO2 | Urine | [19-21] |
| Alanine | 90.0550 | 0.69 | 15% | C3H7NO2 | Urine | [19] |
| Tyrosine | 182.0812 | 1.48 | 24% | C9H11NO3 | Faecal water | [22,23] |
| Isoleucine | 132.1019 | 1.72 | 28% | C6H13NO2 | Faecal water | [22,24] |
| Leucine | 132.1019 | 1.87 | 43% | C6H13NO2 | Faecal water | [22,24] |
| Tryptophan | 205.0972 | 5.35 | 18% | C11H12N2O2 | Urine | [18,25] |
| Lactic acid | 91.0390 | 6.54 | 24% | C3H6O3 | Faecal water | [22] |
| Hippurate | 180.0655 | 6.61 | 21% | C9H9NO3 | Urine | [18,19,21,26] |
| Creatinine | 114.0662 | 0.67 | 9% | C4H7N3O | Urine | [18,20] |
| Mannitol | 183.0863 | 0.60 | 26% | C6H14O6 | Urine | [18] |
| Carnitine | 162.1125 | 0.64 | 9% | C7H15NO3 | Urine | [18,20] |
| Valine | 118.0863 | 0.64 | 15% | C5H11NO2 | Faecal water | [22,24] |
| Glucose | 181.0707 | 0.66 | 31% | C6H12O6 | Faecal water<br>Urine | [27]<br>[28,29] |
| Allantoin | 159.0513 | 0.65 | 15% | C4H6N4O3 | Urine | [18,20] |
| Trigonelline | 138.055 | 0.67 | 10% | C7H7NO2 | Urine | [18,19,26] |
| Acetoacetate | 103.039 | 0.76 | 20% | C4H6O3 | Urine | [19] |
| Glycylproline | 173.0921 | 0.85 | 41% | C7H12N2O3 | Urine | [18] |
| Asparginine | 133.0608 | 0.83 | 33% | C4H8N2O3 | | |
| Methionine | 150.0583 | 1.07 | 15% | C5H11NO2S | Urine | [20] |
| Hypoxanthine | 137.0458 | 1.19 | 29% | C5H4N4O | Urine | [18] |
| Glutamine | 147.0764 | 1.27 | 26% | C5H10N2O3 | Urine | [18] |
| Proline | 116.0706 | 1.45 | 26% | C5H9NO2 | Serum | [18,20] |
| Phenylalanine | 166.0863 | 3.65 | 52% | C9H11NO2 | Urine | [18,20] |
| Xylose | 151.0601 | 4.11 | 37% | C5H10O5 | Urine | [18] |
| Succinate | 119.0339 | 6.91 | 20% | C4H6O4 | Urine | [18,19,26,29] |
| Aspartic acid | 134.0302 | 7.06 | 25% | C4H7NO4 | Faecal water | [22] |
| Lactose | 343.1235 | 7.35 | 50% | C12H22O11 | Urine | [18] |
| GPCho(16:0/0:0) | 496.3398 | 16.34 | 12% | C24H50NO7P | Colonic tissue, colonocytes, plasma | [30,31] |
| GPCho(18:0/0:0) | 524.3711 | 17.29 | 11% | C26H54NO7P | Colonic tissue, colonocytes, plasma | [30,31] |

[a] Retention time (RT), [b] Quality control (QC), [c] Relative standard deviation (RSD) calculated by dividing the standard deviation by the mean in the current study, [d] References that these metabolites were found in urine are reported; otherwise studies in faecal water extracts, serum and colonic tissue are mentioned.

## Supplementary figures

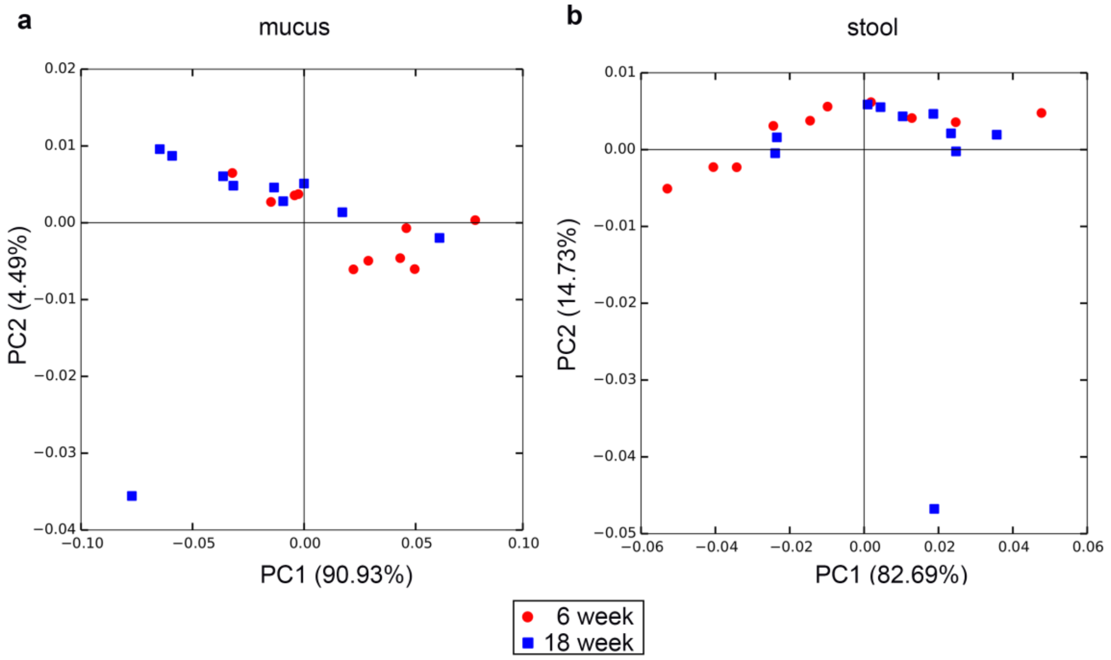


**Supplementary Figure S1. The effect of age on microbial gene functional patterns**. PCoA plot of **(a)** mucus (Adonis test; R2=0.24 P=0.021) and **(b)** stool samples (Adonis test; R2=0.102, P=0.145) based on Bray-Curtis distance of KEGG metabolic pathways. Age had no effect in segregating groups in separate clusters in stool samples, but it appears to have a role in mucus.

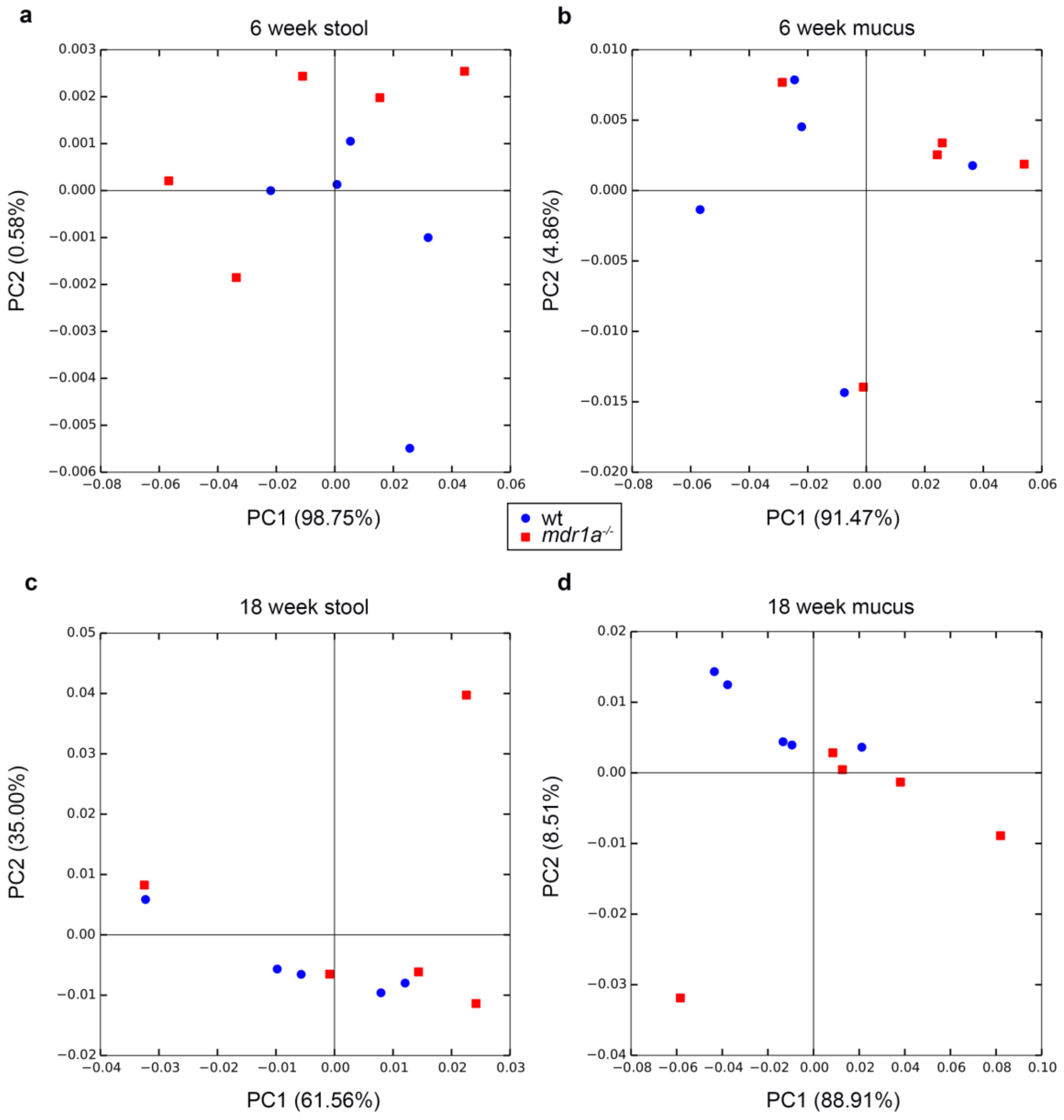


**Supplementary Figure S2. Impact of genotype on microbial gene functional patterns.** PCoA plots using Bray-Curtis distance metric revealed no clustering based on genotype in stool microbial communities at **(a)** 6 (Adonis test; R2=0.079, P=0.42) or **(c)** 18 weeks (Adonis test; R2=0.095, P=0.397) or in mucus-associated bacteria at **(b)** 6 weeks (Adonis test; R2=0.191, P=0.227) or **(d)** 18 weeks (Adonis test; R2=0.188, P=0.178).

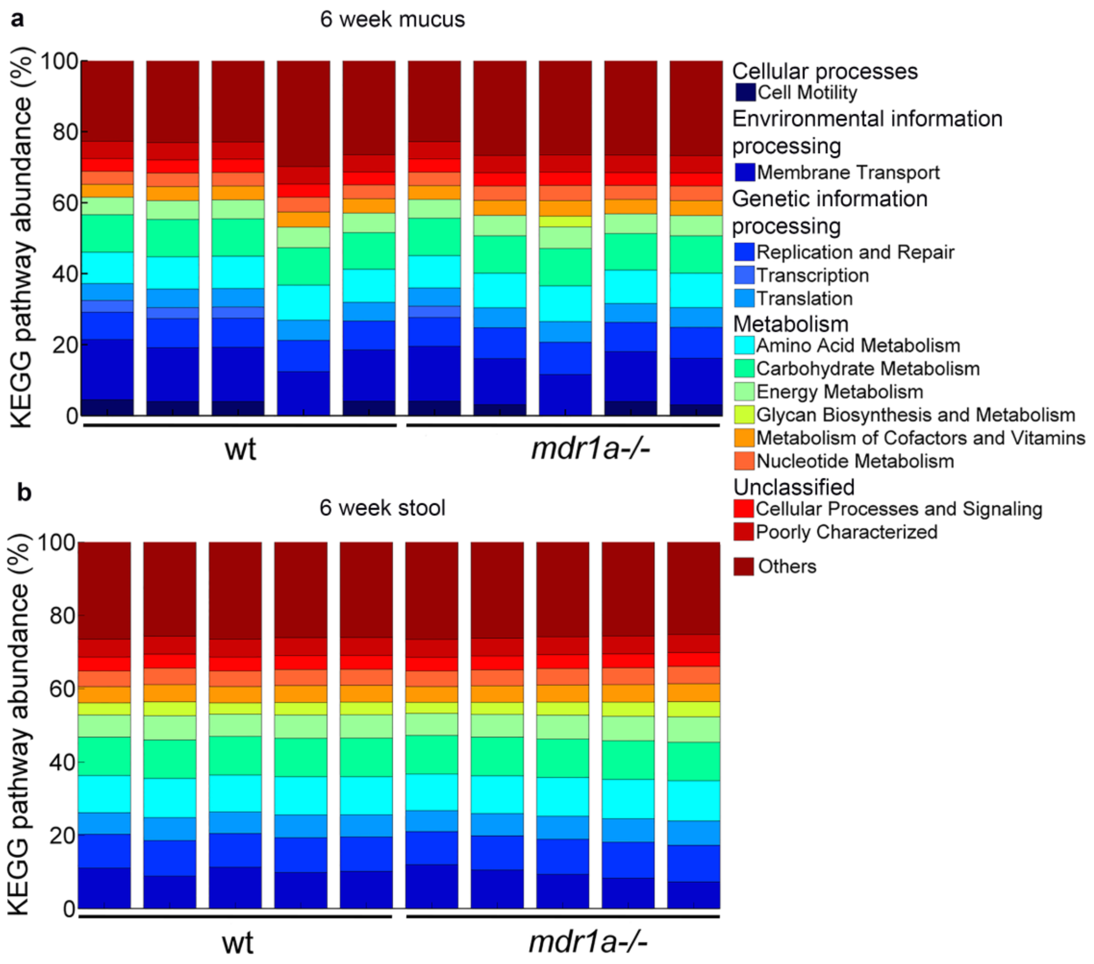


**Supplementary Figure S3. Similarity of the microbial functional potential in WT and *mdr1a*$^{-/-}$ mice before the onset of inflammation.** Relative abundance of KEGG metabolic pathways in **(a)** mucus and **(b)** stool microbial communities at 6 weeks. The category ‘others’ represents KEGG pathways with abundance below 0.3%.

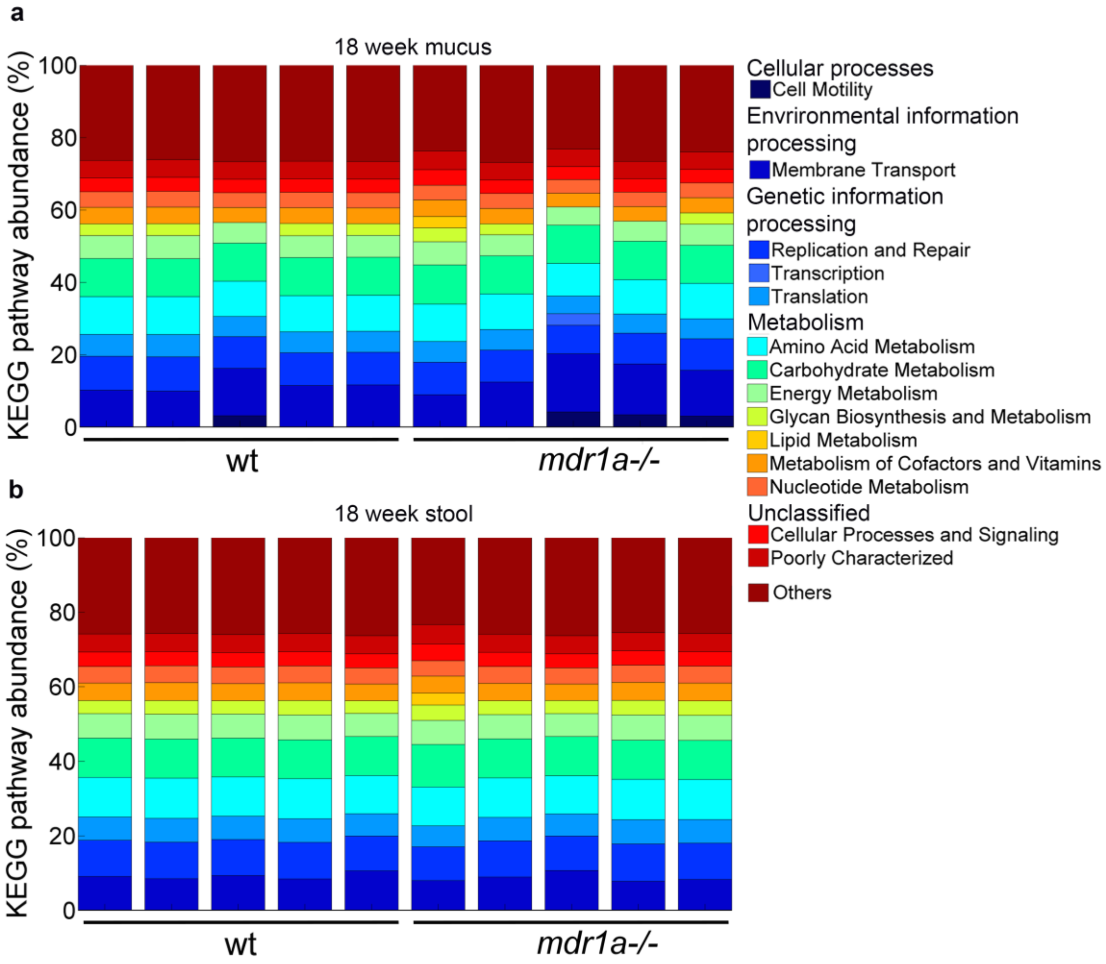


**Supplementary Figure S4. Resilience of the microbial functional potential in WT and colitis prone *mdr1a*$^{-/-}$ mice during colitis onset.** Relative abundance of KEGG metabolic pathways in **(a)** mucus and **(b)** stool microbial communities at 18 weeks. The category ‘others’ represents KEGG pathways with abundance below 0.3%.

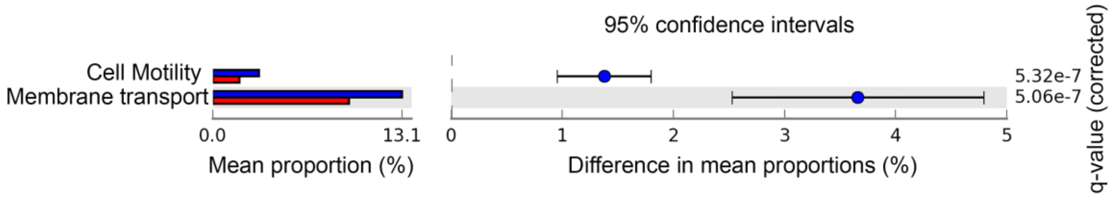


**Supplementary Figure S5. Differences in KEGG pathways from mucus and stool microbial communities.** The pathways at level 2 subsystems are shown. Pathways overrepresented in the mucus (blue) or stools (red) are indicated. Corrected p-values were calculated using Benjamini–Hochberg false discovery rate (FDR). Effect size measures (difference between proportions) and their 95% confidence intervals are shown.

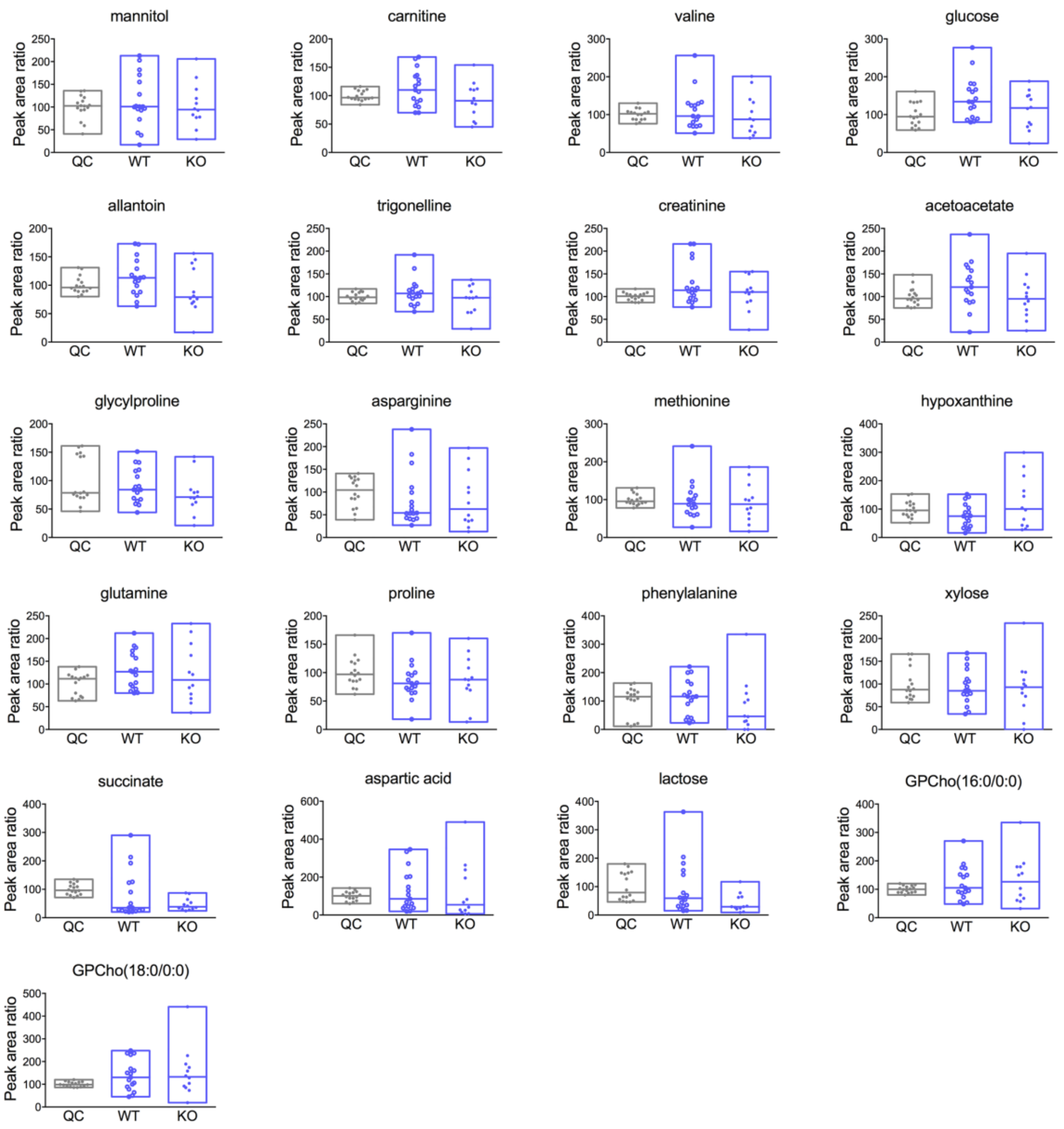


**Supplementary Figure S6. Relative amounts of known IBD reported marker metabolites in urinary samples from WT and *mdr1a*$^{-/-}$ mice at 18 weeks.** No differences were identified in the relative concentrations of metabolites in WT and KO samples during onset of signs of inflammation at 18 weeks. Creatine was used as an internal control in these calculations. N=17 for WT and N=12 for *mdr1a*$^{-/-}$ mice. The median is shown as a line and bars capture the minimum and maximum. Unpaired t-test or Mann Whitney test were applied for comparison between WT and KO samples depending on whether the data were normally distributed or not.

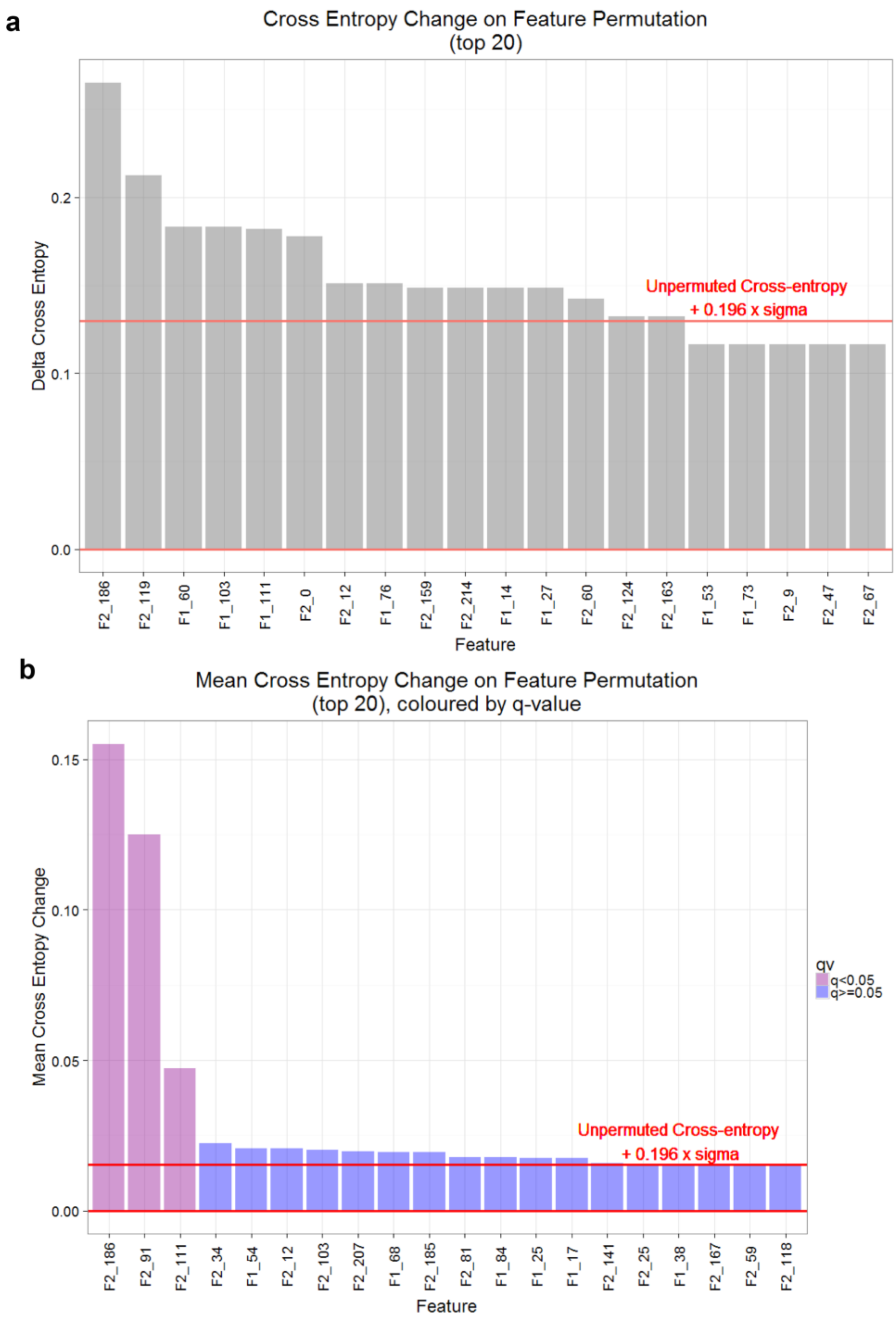


**Supplementary Figure S7. Feature permutation for the identification of discriminatory mass ions responsible for differential classification based on genotype. (a)** Features were permuted using mass ion data from all samples as input, and cross entropy was calculated using a random forest (RF) classifier. **(b)** Features with a difference between mean cross entropy of permuted and unpermuted data greater than 1.96 * sigma were regarded as significant. The Storey multiple correction method was applied.

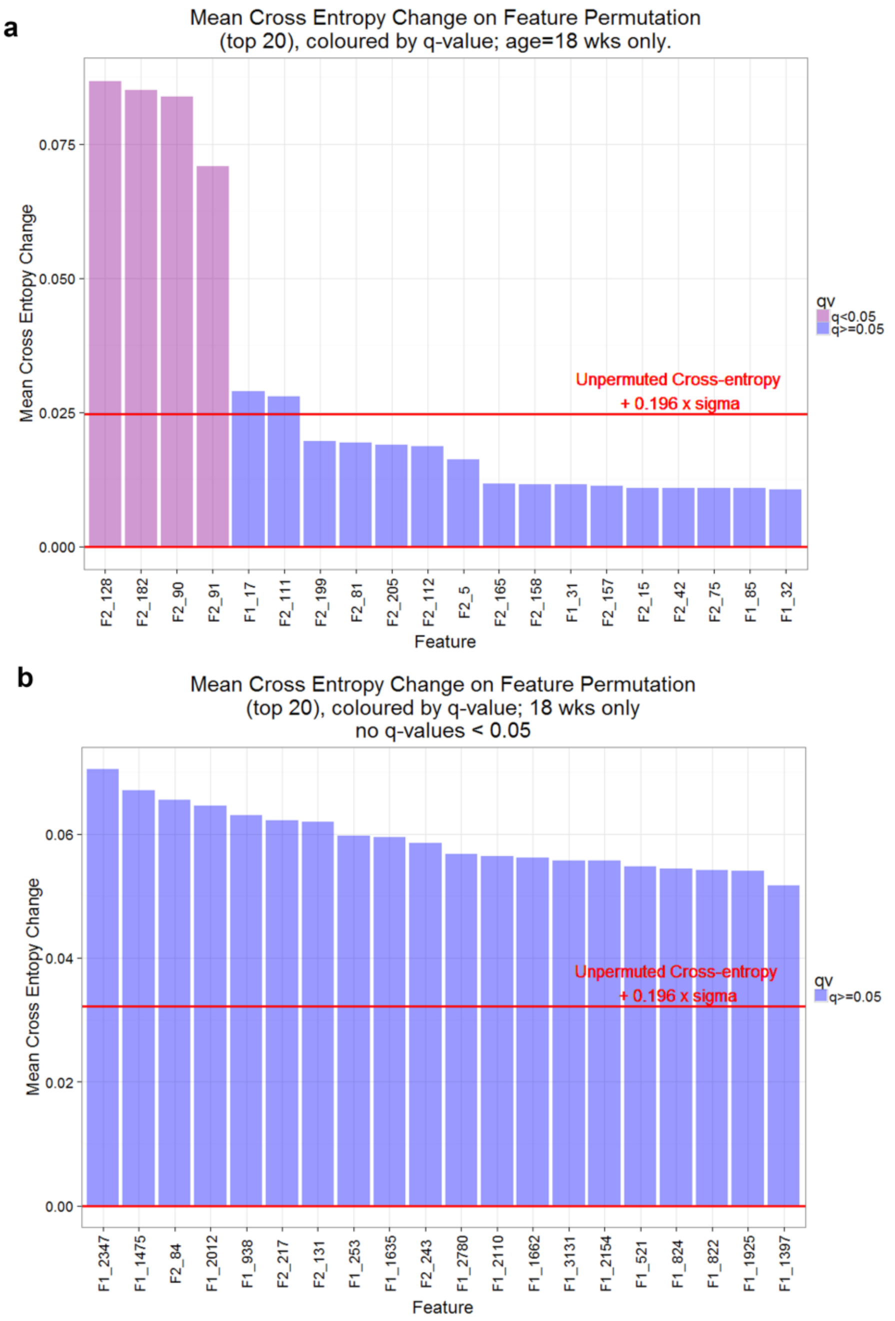


**Supplementary Figure S8. Feature permutation for the detection of mass ions contributing to separation of metabolite profiles of WT and *mdr1a*$^{-/-}$ mice.** Features were permuted using mass ion data from 18-week old samples only as input and cross entropy was calculated using a RF classifier. Permutation results are shown from 18-week samples **(a)** of the initial dataset and **(b)** of the re-processed dataset to correct for noise errors. Features with a difference between mean cross entropy of permuted and unpermuted data greater than 1.96 * sigma were regarded as significant. The Storey multiple correction method was applied.

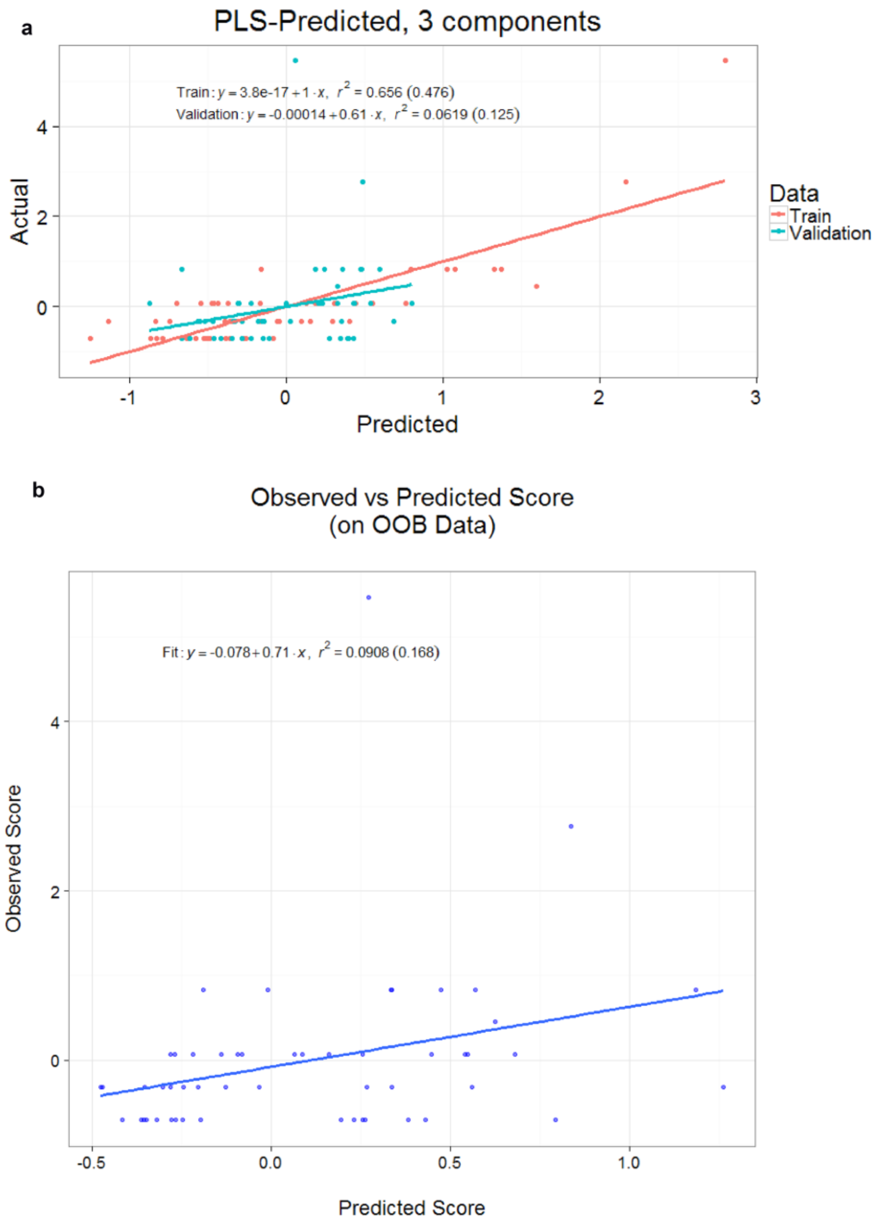


**Supplementary Figure S9. Changes in metabolite profiles were not related to intestinal inflammation.** The PLS and RF regression plots show that the predicted colitis scores based on the pattern of urinary metabolites does not correlate with the actual colitis score. **(a)** PLS plot; actual versus predicted score for training data and leave-one-out cross validation data with linear regression fit; also showing fit equations with squared Pearson correlation coefficient and (squared Spearman correlation coefficient). **(b)** RF plot, colitis score predictions using "Out-of-Bag" data with linear fit, fit equation and squared correlation coefficients. The low R2 values for both techniques indicate poor predictive performance.

## References


1 Glymenaki, M. *et al.* Compositional changes in the gut mucus microbiota precede the onset of colitis-induced inflammation. *Inflammatory bowel diseases* (2017).

2 Friswell, M. K. *et al.* Site and strain-specific variation in gut microbiota profiles and metabolism in experimental mice. *PloS one* **5**, e8584, doi:10.1371/journal.pone.0008584 (2010).

3 Hunter, S. *et al.* EBI metagenomics--a new resource for the analysis and archiving of metagenomic data. *Nucleic acids research* **42**, D600-606, doi:10.1093/nar/gkt961 (2014).

4 Caporaso, J. G. *et al.* QIIME allows analysis of high-throughput community sequencing data. *Nature methods* **7**, 335-336, doi:10.1038/nmeth.f.303 (2010).

5 Edgar, R. C. Search and clustering orders of magnitude faster than BLAST. *Bioinformatics* **26**, 2460-2461, doi:10.1093/bioinformatics/btq461 (2010).

6 McDonald, D. *et al.* An improved Greengenes taxonomy with explicit ranks for ecological and evolutionary analyses of bacteria and archaea. *The ISME journal* **6**, 610-618, doi:10.1038/ismej.2011.139 (2012).

7 Wang, Q., Garrity, G. M., Tiedje, J. M. & Cole, J. R. Naive Bayesian classifier for rapid assignment of rRNA sequences into the new bacterial taxonomy. *Applied and environmental microbiology* **73**, 5261-5267, doi:10.1128/AEM.00062-07 (2007).

8 Langille, M. G. *et al.* Predictive functional profiling of microbial communities using 16S rRNA marker gene sequences. *Nature biotechnology* **31**, 814-821, doi:10.1038/nbt.2676 (2013).

9 Parks, D. H. & Beiko, R. G. Identifying biologically relevant differences between metagenomic communities. *Bioinformatics* **26**, 715-721, doi:10.1093/bioinformatics/btq041 (2010).

10 Berthold, M. R. *et al.* in *Data Analysis, Machine Learning and Applications: Proceedings of the 31st Annual Conference of the Gesellschaft für Klassifikation e.V., Albert-Ludwigs-Universität Freiburg, March 7–9, 2007* (eds Christine Preisach, Hans Burkhardt, Lars Schmidt-Thieme, & Reinhold Decker) 319-326 (Springer Berlin Heidelberg, 2008).

11 O'Hagan, S. & Kell, D. B. Software review: the KNIME workflow environment and its applications in genetic programming and machine learning. *Genetic Programming and Evolvable Machines* **16**, 387-391, doi:10.1007/s10710-015-9247-3 (2015).

12 Mazanetz, M. P., Marmon, R. J., Reisser, C. B. & Morao, I. Drug discovery applications for KNIME: an open source data mining platform. *Current topics in medicinal chemistry* **12**, 1965-1979 (2012).

13 Ramette, A. Multivariate analyses in microbial ecology. *FEMS microbiology ecology* **62**, 142-160, doi:10.1111/j.1574-6941.2007.00375.x (2007).

14 Barker, M. & Rayens, W. Partial least squares for discrimination. *Journal of Chemometrics* **17**, doi:10.1002/cem.785 (2003).

15 Gromski, P. S. *et al.* A tutorial review: Metabolomics and partial least squares-discriminant analysis--a marriage of convenience or a shotgun wedding. *Analytica chimica acta* **879**, 10-23, doi:10.1016/j.aca.2015.02.012 (2015).

16 Cutler, D. R. *et al.* Random forests for classification in ecology. *Ecology* **88**, 2783-2792 (2007).

17 Breiman, L. Random Forests. *Machine Learning* **45**, 5-32, doi:10.1023/a:1010933404324 (2001).

18 Schicho, R. *et al.* Quantitative metabolomic profiling of serum, plasma, and urine by (1)H NMR spectroscopy discriminates between patients with inflammatory bowel disease and healthy individuals. *Journal of proteome research* **11**, 3344-3357, doi:10.1021/pr300139q (2012).

19 Dawiskiba, T. *et al.* Serum and urine metabolomic fingerprinting in diagnostics of inflammatory bowel diseases. *World journal of gastroenterology : WJG* **20**, 163-174, doi:10.3748/wjg.v20.i1.163 (2014).

20 Schicho, R. *et al.* Quantitative metabolomic profiling of serum and urine in DSS-induced ulcerative colitis of mice by (1)H NMR spectroscopy. *Journal of proteome research* **9**, 6265-6273, doi:10.1021/pr100547y (2010).

21 Williams, H. R. *et al.* Characterization of inflammatory bowel disease with urinary metabolic profiling. *The American journal of gastroenterology* **104**, 1435-1444, doi:10.1038/ajg.2009.175 (2009).

22 Bjerrum, J. T. *et al.* Metabonomics of human fecal extracts characterize ulcerative colitis, Crohn's disease and healthy individuals. *Metabolomics :*

*Official journal of the Metabolomic Society* **11**, 122-133, doi:10.1007/s11306-014-0677-3 (2015).

23 Jansson, J. *et al.* Metabolomics reveals metabolic biomarkers of Crohn's disease. *PloS one* **4**, e6386, doi:10.1371/journal.pone.0006386 (2009).

24 Marchesi, J. R. *et al.* Rapid and noninvasive metabonomic characterization of inflammatory bowel disease. *Journal of proteome research* **6**, 546-551, doi:10.1021/pr060470d (2007).

25 Lin, H. M. *et al.* Metabolomic analysis identifies inflammatory and noninflammatory metabolic effects of genetic modification in a mouse model of Crohn's disease. *Journal of proteome research* **9**, 1965-1975, doi:10.1021/pr901130s (2010).

26 Stephens, N. S. *et al.* Urinary NMR metabolomic profiles discriminate inflammatory bowel disease from healthy. *Journal of Crohn's & colitis* **7**, e42-48, doi:10.1016/j.crohns.2012.04.019 (2013).

27 Le Gall, G. *et al.* Metabolomics of fecal extracts detects altered metabolic activity of gut microbiota in ulcerative colitis and irritable bowel syndrome. *Journal of proteome research* **10**, 4208-4218, doi:10.1021/pr2003598 (2011).

28 Lin, H. M., Edmunds, S. I., Helsby, N. A., Ferguson, L. R. & Rowan, D. D. Nontargeted urinary metabolite profiling of a mouse model of Crohn's disease. *Journal of proteome research* **8**, 2045-2057, doi:10.1021/pr800999t (2009).

29 Murdoch, T. B. *et al.* Urinary metabolic profiles of inflammatory bowel disease in interleukin-10 gene-deficient mice. *Analytical chemistry* **80**, 5524-5531, doi:10.1021/ac8005236 (2008).

30 Lin, H. M., Helsby, N. A., Rowan, D. D. & Ferguson, L. R. Using metabolomic analysis to understand inflammatory bowel diseases. *Inflammatory bowel diseases* **17**, 1021-1029, doi:10.1002/ibd.21426 (2011).

31 Zhang, X. *et al.* Metabolite profiling of plasma and urine from rats with TNBS-induced acute colitis using UPLC-ESI-QTOF-MS-based metabonomics--a pilot study. *FEBS J* **279**, 2322-2338, doi:10.1111/j.1742-4658.2012.08612.x (2012).
